# The Caregiver Health Effects of Caring for Young Children with Developmental Disabilities: A Meta-analysis

**DOI:** 10.1007/s10995-020-02896-5

**Published:** 2020-02-11

**Authors:** Sarah C. Masefield, Stephanie L. Prady, Trevor A. Sheldon, Neil Small, Stuart Jarvis, Kate E. Pickett

**Affiliations:** 1 Department of Health Sciences, University of York, Heslington, York, YO10 5DD UK; 2 Faculty of Health Studies, University of Bradford, Bradford, UK

**Keywords:** Systematic review, Meta-analysis, Caregiver, Health, Developmental disabilities

## Abstract

**Objectives:**

Mothers of school age and older children with developmental disabilities experience poorer health than mothers of typically developing children. This review assesses the evidence for the effect on mothers’ health of caring for young children with developmental disabilities, and the influence of different disability diagnoses and socioeconomic status.

**Methods:**

Medline, EMBASE, PsycINFO and CINAHL were searched. Studies measuring at least one symptom, using a quantitative scale, in mothers of preschool children (0–5 years) with and without a diagnosed developmental disability were selected. Random effects meta-analysis was performed, and predictive intervals reported due to high expected heterogeneity.

**Results:**

The meta-analysis included 23 estimates of association from 14 retrospective studies for the outcomes of stress (n = 11), depressive symptoms (n = 9), general health (n = 2) and fatigue (n = 1). Caring for a child with a developmental disability was associated with greater ill health (standardised mean difference 0.87; 95% predictive interval − 0.47, 2.22). The largest association was for mixed developmental disabilities (1.36; − 0.64, 3.36) and smallest for Down syndrome (0.38; − 2.17, 2.92). There was insufficient socioeconomic information to perform subgroup analysis. The small number of studies and data heterogeneity limited the precision of the estimates of association and generalizability of the findings.

**Conclusions for Practice:**

Mothers of young children with developmental disabilities may have poorer health than those with typically developing children. Research is needed to identify whether the relationship is causal and, if so, interventions that could reduce the negative effect of caregiving.

**Electronic supplementary material:**

The online version of this article (10.1007/s10995-020-02896-5) contains supplementary material, which is available to authorized users.

## Significance

Maternal ill health adversely affects mother–child attachment and child emotional and social development. This review illustrates that, during the preschool period, mothers of children with developmental disabilities may have worse health than other mothers across a range of psychological and physical symptoms. This review showed significant variation in the association between caregiving and ill health during the preschool period which was not explained by children’s different disability diagnoses. This variation may be due to common stressors during the preschool period that could be targeted to limit the adverse effect of caregiving on health.

## Objectives

Developmental disabilities are long term physiological impairments which significantly affect a child’s ability to perform activities of daily living, such as independent feeding, communicating and mobilising (World Health Organization and Unicef 2012). The estimated prevalence of intellectual and developmental disabilities in high income countries ranges from 1 to 4% of children (Maulik et al. 2011; Roeleveld et al. 1997); although prevalence may be as high as 15% for the US (Boyle et al. 2011). Estimates may differ depending on the specific disabling conditions included, which are often selected for pragmatic reasons (e.g. multi-country data are available) (Global Research on Developmental Disabilities Collaborators 2018). Classification typically includes autism spectrum disorders (hereafter shortened to autism) and Down syndrome, sometimes vision/hearing loss and epilepsy. Estimates can also vary if a disability severity threshold is used (e.g. only children with functional impairment in more than one skill domain are included) (Horridge et al. 2016). No studies have estimated prevalence exclusively for the preschool age group.

Studies provide substantial evidence that parents of school-age and older children with developmental disabilities experience elevated levels of stress and depressive symptoms, above that of parents of typically developing children (Plant and Sanders 2007; Singer and Floyd 2006; Smith et al. 2001). Higher rates of general ill health, sleep problems, headaches and musculoskeletal pain have also been found (Fairthorne et al. 2015; Lee et al. 2017; Miodrag et al. 2015; Miodrag and Hodapp 2010). Mothers are typically the primary caregiver, with evidence of worse health outcomes than fathers (Allik et al. 2006; Sloper and Turner 1993). Studies on caregiver health are typically conducted in caregivers of children with cerebral palsy, autism or mixed disability groups (comprised to a large extent of children with these disabilities and Down syndrome), frequently without the inclusion of a typically developing comparison group (Bailey et al. 2007).

Ill health adversely affects mother–child attachment and mothers’ perception of the difficulties and demands of caregiving (Howe 2006; Shonkoff et al. 1992; Witt et al. 2003). The stress-health mechanism, whereby caregivers experience greater stress than parents of typically developing children resulting in poorer health outcomes, has been used to understand the caregiver health relationship (Raina et al. 2004). Stressors include: seeking pediatric assessment and diagnosis; adapting to the caregiver role and its impact on employment; and experiencing disability stigma (Beresford et al. 2007; Warfield et al. 1999). There may be additional physical demands which contribute to caregiver burden, such as assisting with transfers into a wheelchair and undertaking physical therapy.

In the caregiver burden model (Raina et al. 2004), direct relationships between caregiving for a child with any degree of functional impairment and caregiver psychological and physical ill health are modified by different child disability diagnoses and socioeconomic factors. For example, caregiving for a child with autism, severe impairment or behavioral problems have been associated with greater ill health, and socioeconomic advantage with lesser ill health (Chatel Garriot et al. 2014; Plant and Sanders 2007; Roper et al. 2014; Shonkoff et al. 1992).

Advances in medical knowledge and technology have led to the diagnosis of many disabilities before the age of five. Parents report especially high emotional stress during the process of seeking and receiving a disability diagnosis for their child, which usually begins during the preschool period (Graungaard and Skov 2006). However, caregiver health research has largely focused on parents of school-age and older children (De Giacomo and Fombonne 1998; Ward and Soothill 2011). Poor health observed in mother-caregivers of younger children may have implications for the timing of appropriate interventions to assist them.

Every literature review on caregiver health that we have identified includes wide age ranges, with little or no subgroup analysis by age; and many examine health in the mothers of children with specific disability diagnoses e.g. autism (Biswas et al. 2015; Fairthorne et al. 2018; Hayes and Watson 2013; Bekhet et al. 2012; Honey et al. 2005). Meta-analysis may be able to resolve controversies arising from conflicting study findings, yet very few of these reviews included meta-analyses (Sanderson et al. 2007; Singer and Floyd 2006). Outstanding controversies include: whether caregiving for a child with developmental disabilities has a direct adverse influence on mothers’ health; whether the adverse association emerges during the preschool period; and the influence of different specific disability-related factors (Green 2007; Stoneman 2007).

This paper reports a systematic review with meta-analysis to investigate symptoms of ill health in mothers of preschool children with developmental disabilities compared to mothers of typically developing preschool children, and to identify whether disability diagnosis and socioeconomic status (SES) might explain any differences.

## Methods

The PRISMA checklist was used to guide the reporting of the systematic review methods and results (Online Resource) (Moher et al. 2009). The Cochrane Handbook for Systematic Reviews of Interventions 5.1.0 was used for guidance on conducting systematic reviews with meta-analysis (Higgins and Green 2011). The review protocol was not registered.

The databases Medline(OVID), EMBASE(OVID), PsycINFO(OVID) and CINAHL(EBSCO) were searched. The search strategy was developed to retrieve articles on health outcomes in caregivers [including stress—both an indicator of psychological distress and a risk factor for ill health (Schneiderman et al. 2005)] (Table 1). The search strategy used subject headings for generic terms for ill health and disability. Generic and specific key terms for disabilities (e.g. autism) and symptoms of ill health (e.g. fatigue) associated with caregiver burden and identified from scoping the literature were included. The search was limited to observational study designs which were expected to include a typically developing comparison group (BMJ Clinical Evidence 2018). Accordingly, grey literature was not searched and intervention studies, which commonly draw the comparison group from the same population, were excluded. No lower publication date limit was specified.

**Table 1:** The literature search strategy used in Medline, EMBASE and PsycINFO

1.(((mother-carer* or mother carer* or mother caregiver* or mother care-giver* or parent-carer* or parent carer or parent care giver* or parent care-giver* or carer* or care-giver* or caregiver* or care giver* or family caregivers or mother* or parent* or parenting or caring) adj2 (asthma or arthritis or allergies or food allergies or rheumatism or joint pain or joint symptom* or neck pain or neck problem* or back pain or back problem* or migraine* or headache* or diabetes or hypertension or high blood pressure or sinusitis or heart condition* or heart disease or chronic bronchitis or bronchitis or emphysema or sleep problem* or sleep disturbance or sleep deprivation or poor quality of sleep or fatigue or exhaustion or stomach ulcer* or intestinal ulcer* or gastrointestinal problem* or gastrointestinal condition* or pain or stress or low mood or depression or back or neck or stomach or mobility or vision or hearing or sleep or joint or anxiety or depressive symptom* or cold or common cold or cold symptom* or flu or flu symptom* or symptom* or physical health or physical problem* or psychological health or psychosocial problem* or general health or ill-health or ill health or poor health or chronic conditions or mental health or mental health problems or psychological distress or emotional problem*)) or (burden of care or burden of caring or care* burden or caregiver burden or care-giver burden or caregiver strain or care-giver strain or strain" or burden)).mp 2. (((behaviour* or emotion* or conduct or development* or communication or social* or mental health or anti-social or learning or cognition or intellectual or psychomotor or growth or congenital or chronic or speech or mental* or language development or language or motor skills or neurodevelopmental or sensory or rare or complex or childhood-onset or intellectual development or anti-social behaviour or attention deficit hyperactivity or autis* spectrum) adj1 (disorder or problem or need* or behaviour or behavior or disabil* or disabl* or handicap* or impair* or condition or anomal* or abnormalit or retard*) adj2 (child* or infant or newborn or new born or pre-school or preschool or primary school or neonat*)) or (disabled child* or child* with disabilities or child* with disability or handicapped child* or child* with handicap* or impaired child or child with impairment or disabl* infant* or disabl* newborn*)).mp 3. ((cerebral palsy or autis* or Down* syndrome or deaf* or blind* or epilepsy or attention-deficit-hyperactivity-disorder) adj2 (child* or infant or newborn or new born or pre-school or preschool or primary school or neonat*)).mp 4. 2 or 3 5. 1 and 4 6. 5 not (adults with disabilities or disabled adults or disabled parent* or disabled mother or mother with disabilities).mp 7. exp cohort studies/ 8. cohort$.tw 9. controlled clinical trial.pt 10. epidemiologic methods/ 11. limit 10 to year = 1966–1989 12. exp case–control studies/ 13. (case$ and control$).tw 14. or/7–9,11–13 15. 6 and 14	

The asterisks indicate truncation

The article inclusion criteria were: (1) a quantitative scale was used to measure at least one symptom of ill health and the central tendency reported for caregiver and comparison groups; (2) > 50% of the caregiver and comparison groups were mothers; (3) children aged 0–5 (mean age < 5) (studies not describing the children by age were excluded); (4) diagnosed with at least one developmental disability (samples with > 50% with developmental delay, at risk of developmental disability, or unspecified disabilities were excluded); (6) publication in English; (7) study conducted in an OECD country. Studies of children with disability due to traumatic injury or conditions not causally associated with substantial long-term developmental disability were excluded, such as hearing impairment and behavioral problems, unless comorbid with developmental disability.

Titles and abstracts, then the full text of potentially included articles were screened by the lead author according to the inclusion criteria. Feedback on methods, review of data collection tools and analytic output was provided by co-authors. Extracted data (from full text publications) included: information on study characteristics (study design, location, sample size, recruitment); population characteristics (age, ethnicity, marital status, education); ascertainment of case and comparison groups (disability diagnosis, behavioral problems); assessment method and reported outcomes (mean scores and standard deviation; percentage above the clinical cut-off).

The Newcastle–Ottawa Scale was used to evaluate the quality of eligible longitudinal studies (Wells et al. 2017). Each study was assessed in three domains: selection of the exposed group (≤ 4 stars), comparability (≤ 1 stars), and outcome (≤ 3 stars). An adapted version was used for the cross-sectional studies (6 star maximum) (Herzog et al. 2013). For the follow-up criteria, a minimum period of three months was considered long enough for changes in psychological symptoms to be observed (National Institute for Health and Clinical Excellence 2016) and follow up of > 80% of the cohort considered adequate. The star score was converted into a rating of good (≥ 7), fair (2–6) or poor (≤ 1) (McPheeters et al. 2012).

### Analysis

The association measures were reported as standardised mean difference (SMD) (the size of the association relative to the variability of the outcome observed) with 95% confidence intervals (CI). A positive or negative estimate of 0.2 was considered small but not trivial, 0.5 moderate, 0.8 and above a large effect (Durlak 2009). Pooled estimates of the magnitude of the association of caregiving with ill health were calculated using a random effects model.

Missing standard deviations of scores were imputed where possible, either from a study using the same version of the outcome measure or by averaging the standard deviations reported in studies of the same outcome. For longitudinal studies, the outcome measurement from the latest data collection point was used (Higgins and Green 2011).

Heterogeneous data were expected due to different sample sizes, diversity of outcomes and measures, single and mixed disability diagnosis groups. The extent of the variability of SMDs was estimated using the *I*^2^. Subgroup analyses by outcome, disability diagnosis and SES were pre-planned to investigate possible causes of variability. At least three SMDs were required for subgroup analysis (Higgins and Green 2011).

Predictive intervals (PI) were estimated (in addition to confidence intervals) to accommodate the width of the distribution of SMDs across the individual studies. This interval effectively converted the heterogeneity into the same metric as the SMD to give the range within which the association would be situated in a new study with 95% certainty. The predictive intervals facilitated a more realistic interpretation of the association and its clinical implications than a confidence interval (IntHout et al. 2016). The potential for publication bias was evaluated using a funnel plot and Egger regression test (Zwetsloot et al. 2017).

The analysis was conducted using Stata software version 15 (StataCorp LLC 2018).

## Results

### Description of the Included Studies

**Fig. 1:**
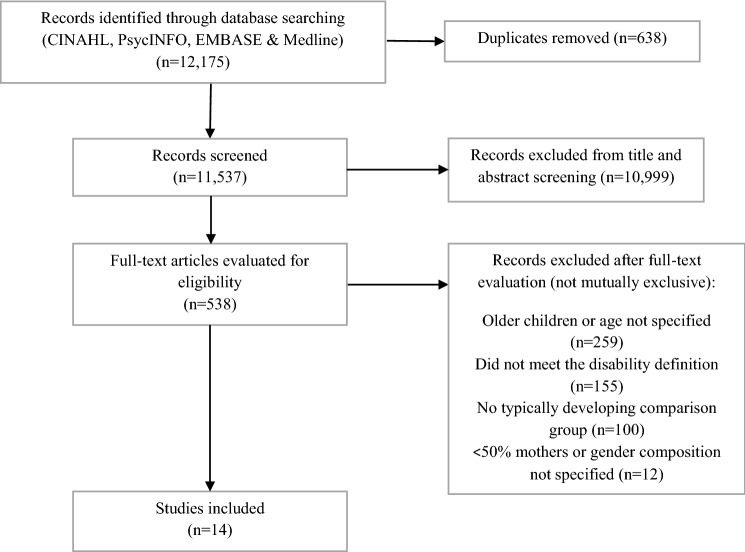
Flow diagram of the process of selection for eligible studies

The search produced 12,175 records. After screening, 14 articles were included (Fig. 1).

Standard deviations were imputed for two outcomes: stress (Roach et al. 1999) and depressive symptoms (Scott et al. 1997); but could not be imputed for psychological distress (Scott et al. 1997), which was only included in the narrative synthesis. One study included three caregiver samples for different disability diagnoses, and six included two health outcomes. Eleven different outcome measures were reported. Half of the included studies were conducted in the USA (n = 7). The mean age of the children in the studies ranged from 9 months to 4.7 years. The studies included groups of children with autism (n = 5), Down syndrome (n = 4), mixed developmental disabilities (n = 4), and cerebral palsy (n = 3). The studies with mixed disability groups included children with the named disabling conditions and other (largely unspecified) disabilities, which were assumed to be significant as the children were all enrolled in programmes for disabled children. The characteristics and study quality assessments are summarised in Table 2.

**Table 2:** Description of the eligible studies with quality ratings (grouped by study design and in alphabetical order)

Study	Country and study design	Outcome	Outcome measure	Disability diagnosis	Caregiver group (n)	Comparison group (n)	Case recruitment method	Comparison recruitment method	Child mean age for cases (in years)a	SES variables^b^	Quality rating scores (n/total; quality rating)
Eisenhower et al. (2005)	USA, longitudinal	Depressive; symptoms Stress	CES depression scale; Family impact questionnaire	Autism Down syndrome Cerebral palsy	14 12 10	136	Disability services	Preschools/day care centres	2.9 (SD 0.26)	Education, ethnicity, employment, income	3/8 Fair
Gowen et al. (1989)	USA, longitudinal	Depressive symptoms	CES depression Scale	Mixed developmental disabilities	21	20	Intervention programmes	Birth records	2.25	Education, ethnicity, SES	4/8 Fair
Jeans et al. (2013)	USA, longitudinal	Depressive symptoms; Stress	CES depression scale; Parenting stress index (1990)	Autism	100	8500	Selected from pre-existing cohort	Same cohort	4 (SD not provided)	Ethnicity	5/8 Fair
Laxman et al. (2015)	USA, longitudinal	Depressive symptoms	CES depression scale	Autism	50	2900	Selected from pre-existing cohort	Same cohort	4 (SD not provided)	Ethnicity	6/8 Fair
Norlin and Broberg (2013)	Sweden, longitudinal	Depressive symptoms; Stress	Beck depression inventory II (revised); Family impact questionnaire	Mixed developmental disabilities	58	182	Disability services	Birth records	4.42 (SD 2.32); comparison 4.44 (1.71)	Education	3/8 Fair
Dyson (1991)	Canada & USA, cross-sectional	Stress	Questionnaire on resources and stress	Mixed developmental disabilities	55	5	Disability services	Preschools, day-care centres, and primary grades	4.4 (SD 1.4); comparison 4.3 (1.7)	Education, ethnicity, SES	3/6 Fair
Eker and Tüzün (2004)	Turkey, cross-sectional	General health	Medical outcomes 36-item short form	Cerebral palsy	40	44	Health centre (inpatient)	Health centre (out-patient services)	4.7 (SD not provided)	Education, employment	3/6 Fair
Giallo et al. (2013)	Australia, cross-sectional	Fatigue	Fatigue assessment scale	Autism	50	1122	Parent support groups and disability services	A community sample	4.20 (SD 1.26)	Education, employment	1/6 Poor
Glenn et al. (2009)	UK, cross-sectional	Stress	Parenting stress index (1995)	Cerebral palsy	80	460	Disability services	N/S (Recruited in the USA)	1.6 (SD 0.74); comparison 2	Education, ethnicity, SES	2/6 Fair
Hedov et al. (2002)	Sweden, cross-sectional	Stress	Parent perception inventory	Down syndrome	86	87	N/S	Birth records	4.7 (SD not provided)	Education, employment	3/6 Fair
Oelofsen and Richardson (2006)	UK, cross-sectional	General health; Stress	Heath perceptions questionnaire; Parenting stress index (1995)	Mixed developmental disabilities	59	45	Disability services	Local preschools & matched by postcode	3.66 (SD 0.76); comparison 3.64 (0.78)	Ethnicity, SES	3/6 Fair
Quintero and McIntyre (2010)	USA, cross-sectional	Depressive symptoms; Stress	CES depression scale Parenting daily hassles scale	Autism	20	23	Intervention programmes	Recruitment via the cases	4.35 (SD 1.12); comparison 3.72 (0.71)	Education, ethnicity, employment, income	2/6 Fair
Roach et al. (1999)	USA, cross-sectional	Stress	Parenting stress index (1995)	Down syndrome	41	58	Regional research database	Birth announcements in local newspapers	3.04 (SD 0.89); comparison 2.43 (1.14)	Education, employment, SES	2/6 Fair
Scott et al. (1997)	Canada, cross-sectional	Depressive symptoms	Beck depression inventory	Down syndrome	188	128	Intervention programmes	Recruitment via the cases	1.21; comparison 1.17 (SD not provided)	Income	2/6 Fair

*CES Depression Scale* Center for Epidemiologic Studies Depression Scale

^a^For longitudinal studies, child age is given for the data collection point of the mean outcome used in the analysis. The age is the same for case and comparison groups unless otherwise stated

^b^*SES* socioeconomic status

Data on SES were inconsistently collected and reported. For example, education was reported in one study as the mean number of years and in another as the percentage of mothers with different levels of educational attainment. Five studies reported the SES distribution of the sample, but none reported the sample representativeness (Glenn et al. 2009; Jeans et al. 2013; Laxman et al. 2015; Oelofsen and Richardson 2006; Roach et al. 1999). None reported the outcome disaggregated by SES. Where interpretable, over half of the studies had socioeconomically advantaged maternal cohorts (n = 6/9), varying with disability diagnosis in one study (Eisenhower et al. 2005).

In every study, the disability diagnosis reported at recruitment (parent-reported or disability service/database record) was accepted without independent verification. None of the studies reported how child typical development in the comparison groups was ascertained. Only one (Eker and Tuzun 2004), reported health assessment in the comparison group children which might increase parent burden, such as asthma or diabetes.

Thirteen studies (n = 13/14; 92.9%) received a quality rating of fair (Table 2). Longitudinal analyses (n = 5/14) received 3–6 stars (x/8), mean 4.2 and the cross-sectional analyses (n = 9/14) 1–3 stars (x/6), mean 2.3. Major factors that compromised study quality were: (1) unknown representativeness of the caregivers due to convenience sampling; (2) inadequate description of comparison group comparability; and (3) failure to assess whether the health outcome preceded the start of caregiving.

### Results of the Meta-analyses

**Fig. 2:**
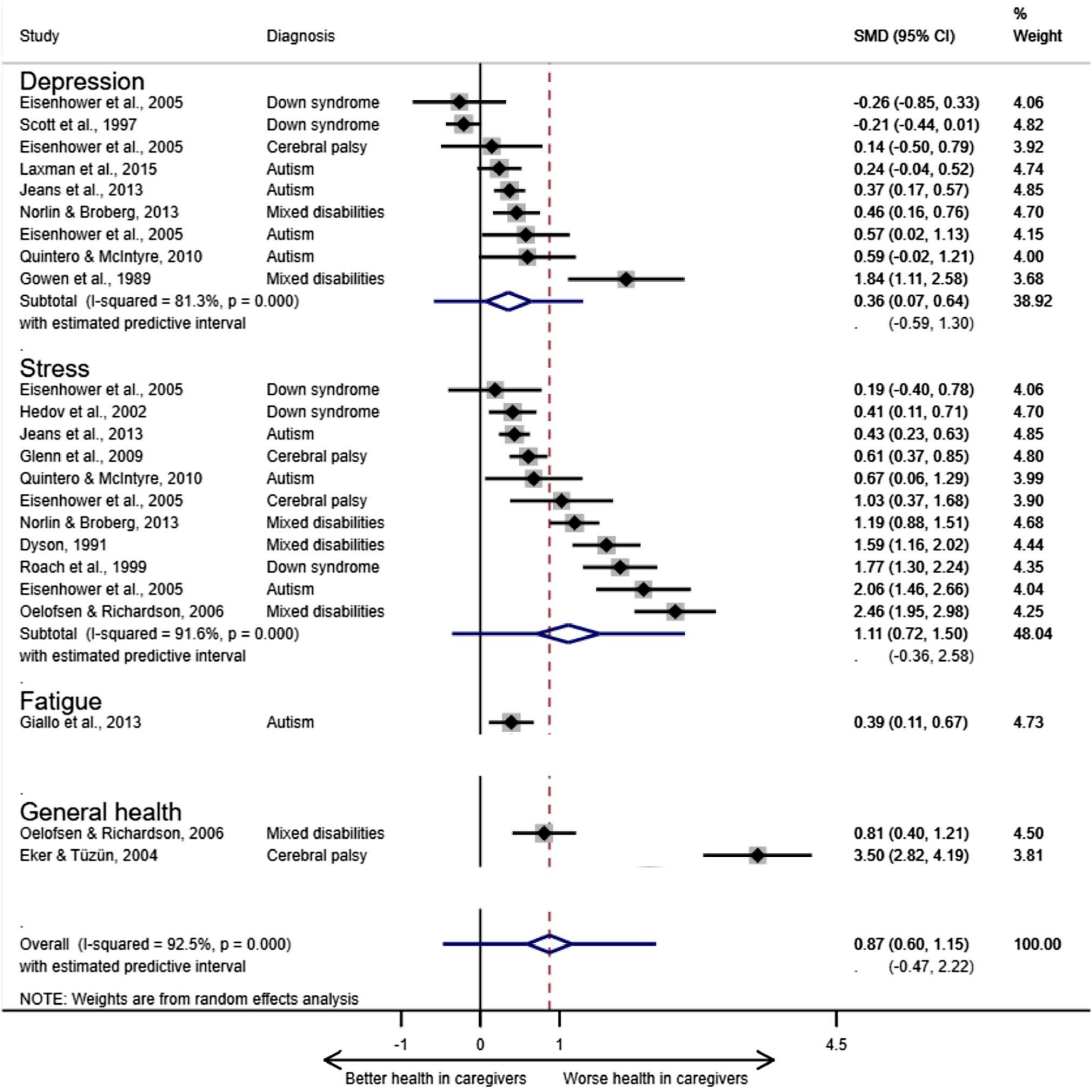
Relationship of caregiving for preschool children with developmental disabilities to ill health by symptom and overall. ^a^Pooled estimates and their 95% CIs are depicted as a diamond. The error bars on the diamond illustrate the predictive interval for the pooled estimate. *SMD*, standardised mean difference (the size of the association). % weight, the contribution of each study to the pooled estimate in the random effects model based on sample size. ^b^The pooled estimates for fatigue and general health subgroups are not displayed as a minimum of three effect sizes was required for the analysis

In total, 23 SMDs from 14 studies were included in the meta-analysis: 12 from longitudinal and 11 cross-sectional study designs (Fig. 2). The outcomes were stress (n = 9), depressive symptoms (n = 7), general health (n = 2), and fatigue (n = 1). Measures of the symptom in parents or the adult population were used in 12 studies (Eisenhower et al. 2005; Norlin and Broberg 2013). The other two studies used the Family Impact Questionnaire to compare stress in caregivers compared with other parents. This measure was designed to address the inherent bias in the assessment of parenting stress using tools that assume learning disabilities and behavioral problems (common in children with developmental disabilities) are causes of stress (Baker et al. 2003). Five of the included studies used measures with this bias (Questionnaire on Resources and Stress, and Parenting Stress Index) (Jeans et al. 2013; Dyson. 1991; Glenn et al. 2009; Oelofsen and Richardson 2006; Roach et al. 1999).

**Fig. 3:**
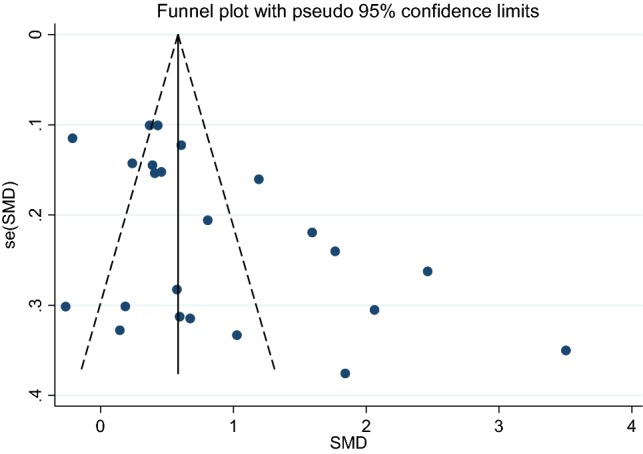
Funnel plot to assess small study bias in the meta-analysis. *Se(SMD)*, standard error of the standardised mean difference

The pooled estimate for the combined outcomes (n = 23) was large (0.87; 95% confidence interval (CI) 0.60, 1.15) but with high uncertainty reflected in the wide predictive interval that included zero (− 0.47, 2.22). The high coefficient for the Egger test suggested the possibility of publication bias (Egger β 4.74; CI 1.40, 8.07). The substantial asymmetry of the funnel plot illustrated the influence of small study size on the precision of the effect sizes (Fig. 3), with a trend for larger study size (plotted as variability) to be associated with lower SMD. As the variability increased, so did the distance from the pooled estimate.

A large adverse relationship of caregiving to stress (1.11; CI 0.72, 1.50) was found and a small-moderate adverse relationship of caregiving to depressive symptoms (0.36; CI 0.07, 0.64). However, the predictive intervals for both symptoms included zero (stress − 0.36, 2.58; depressive symptoms − 0.59, 1.30).

**Fig. 4:**
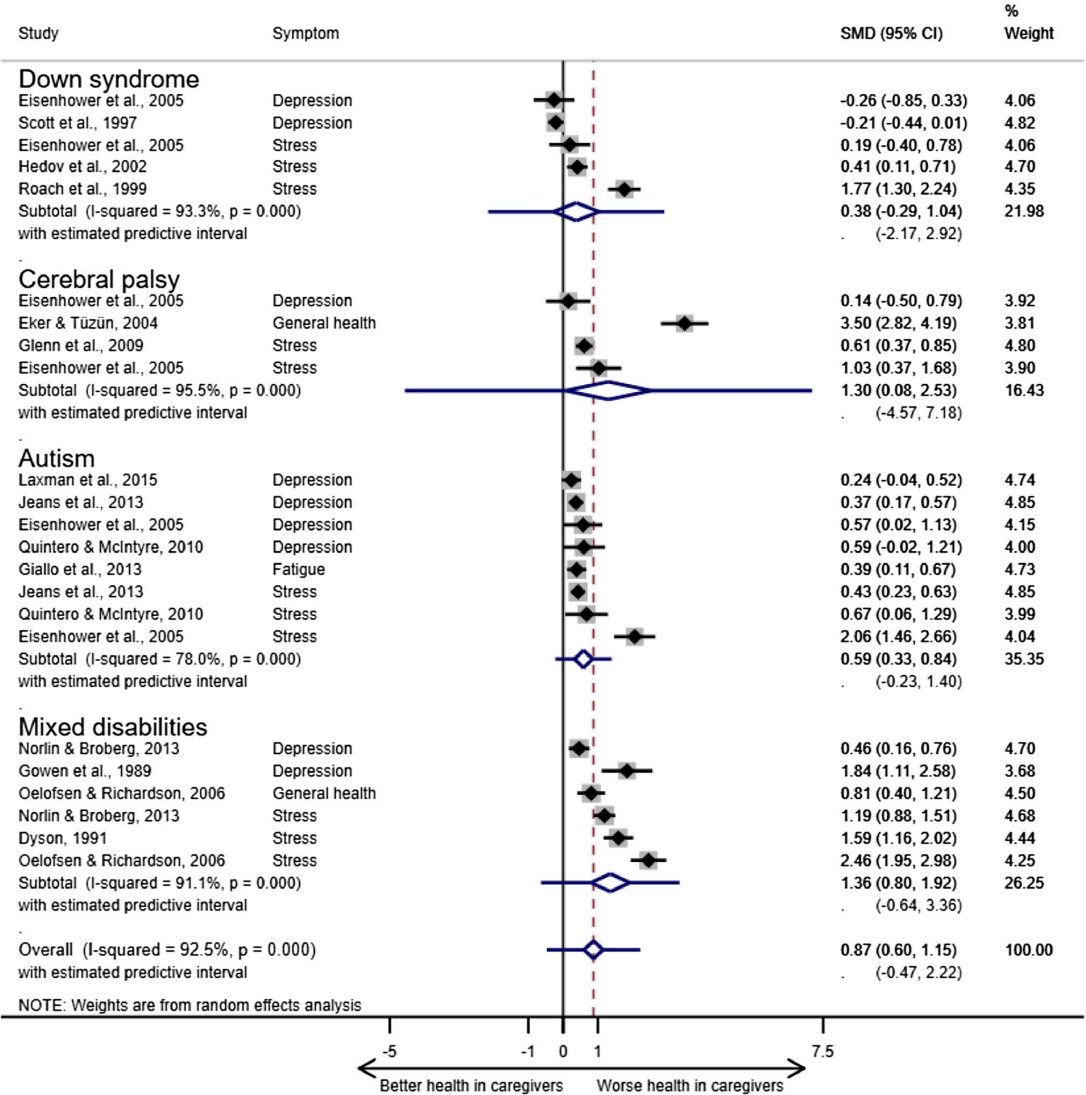
Relationship of child disability diagnosis (mixed disabilities, cerebral palsy, autism and Down syndrome) to caregiver health outcomes. ^a^Pooled estimates and their 95% CIs are depicted as a diamond. The error bars on the diamond illustrate the predictive interval for the pooled estimate. *SMD*, standardised mean difference (the size of the association). % weight, the contribution of each study to the pooled estimate in the random effects model based on sample size

Each disability diagnosis subgroup had an adverse relationship to caregiver ill health, but the predictive intervals (PI) included zero (Fig. 4). The largest pooled estimate was for mixed developmental disabilities (1.36; CI 0.80, 3.36; PI − 0.64, 3.36) and the smallest for Down syndrome (0.38; CI 0.29, 1.04; PI − 2.17, 2.92). The two studies with SMDs below the line of no association (< 0) were Down syndrome samples.

There was high heterogeneity (I^2^ ≥ 78%) for every outcome and diagnosis subgroup, which affected the precision of the estimate that could be made for a new study. For example, compared with our pooled estimate with confidence interval, the true magnitude of the association between caregiving and ill health (based on the predictive interval) may range from none (better health in caregivers than other mothers) to greater (much poorer health in caregivers).

Two extreme SMDs were observed but not excluded (Eker and Tuzun 2004; Gowen et al.^1989^), as these potential outliers may be accurate data points illustrating diversity rather than measurement error, for example.

The inadequacies of the SES information reported in the studies prevented subgroup meta-analysis.

## Discussion

### Maternal Health Outcomes

Our findings support the theorised adverse relationship between caregiving and health during the preschool period. Most of the evidence was for maternal stress and depressive symptoms, very few studies were found for other symptoms. However, the extent to which these symptoms exceeded any clinical threshold was unclear, which might explain variation in outcomes.

These findings are consistent with those of other reviews (narrative and meta-analyses), which all identified greater ill health in more parents of children with developmental disabilities than parents of typically developing children (Fairthorne et al. 2015; Lee 2013; Miodrag et al. 2015; Miodrag and Hodapp 2010; Bailey et al. 2007; Singer and Floyd 2006).

#### Depressive Symptoms

Singer and Floyd’s review (2006) estimated a small-moderate detrimental association between caregiving for children with developmental disabilities (diagnosed before the age of 21 and mostly above 5 years) and symptoms of depression (n = 18 studies; weighted association 0.39; CI 0.31, 0.47). Their pooled estimate (with nonsignificant heterogeneity) was slightly greater and more precise than ours (0.36; CI 0.07, 0.64). Our results provide evidence that the adverse relationship of caregiving to depressive symptoms probably emerges during the child’s preschool years.

#### Stress

No meta-analyses have previously been performed for stress in mothers of children with and without developmental disabilities. Hayes and Watson (2013) found a large adverse association between caregiving for children with autism (of any age, including three studies with an average child age or range below five) and parenting stress (n = 15 studies; 1.58; CI 1.16, 2.0). Our results are consistent with theirs as we show greater stress in caregivers than other mothers during the preschool period (1.11; 95% CI 0.72, 1.50).

#### Physical and General Ill-Health

Using the Parenting Stress Index health sub-domain (Abidin 2017), Miodrag et al. (2015) found evidence of an association between caregiving for children of any age with intellectual disabilities and chronic conditions and poorer parental physical health (0.39; 95% CI 0.23, 0.55). Some of the stress and depressive symptom indices in our review had physical health components, and the general health and fatigue outcomes reported a greater physical than psychological symptom weighting (Giallo et al. 2013; Eker and Tunzen 2004; Oelofsen and Richardson 2006). There was evidence of poorer caregiver health in all these studies (SMD > 0), but sub-group meta-analysis could not be performed due insufficient data.

### Disability Diagnosis

In our meta-analysis, an association between adverse caregiver health and each child diagnostic group was found. Other studies consistently find that mothers of children with autism experience greater stress and depressive symptoms than mothers of children with other developmental disabilities (Sanders and Morgan 1997; Valicenti-McDermott et al. 2015). In the studies included in our review, higher stress and depressive symptom scores were reported for autism than other specific (but not mixed) disability groups. However, heterogeneity remained high in each subgroup, contrary to meta-analyses in single disability groups of older children (Hayes and Watson 2013; Singer and Floyd 2006).

The association of the greatest magnitude was for mixed developmental disabilities, which could reflect a common experience of high stress in caregivers during the preschool period as they seek and receive their child’s diagnosis and adjust to the implications for their lives (Beresford et al. 2007). Alternatively, other disability factors might have a stronger association with caregiver stress during this period than the specific diagnosis, such as child behavioral problems and disability severity (Biswas et al. 2015; Neely-Barnes and Dia 2008; Plant and Sanders 2007).

### Socioeconomic Status

SES has been identified as an influencing factor in caregiver ill health (Emerson 2003; Smith et al. 2001). Miodrag et al. (2015) identified modifying effects of educational attainment, ethnicity and marital status on the size of the adverse relationship of caregiving for children with intellectual disabilities and chronic conditions to physical ill health.

Here, the insufficiency of the data prevented assessment of the influence of SES on caregiver health. Both SMDs with unexpected findings (better health in caregivers) were in samples of children with Down syndrome (n = 5). This possibility is due to the so called ‘Down syndrome advantage’ (Corrice and Glidden 2009). High education is associated with pregnancy in mothers aged 35 and over, who have a raised risk for Down syndrome and have higher average SES than families of children with other developmental disabilities (due to high education) (Stoneman 2007). Accordingly, the apparent better health of caregivers of children with Down syndrome compared with other caregivers is largely attributable to the protective effect of socioeconomic advantage (and possibly to fewer child behavioral problems) (Emerson et al. 2006).

### Heterogeneity

High heterogeneity was present in all the meta-analyses. Small study publication bias was present which might lead to an overestimate of the magnitude of the association but is unlikely to be the sole explanation for the asymmetry highlighted by the Egger et al. test (1997). Other factors may explain the heterogeneity, such as child behavior, maternal health status prior to the child’s birth, social support or differences in the maternal and child populations (Demir et al. 2008; Dunst et al. 1986). Insufficient information was provided in the studies to explore these possibilities.

An association between caregiving for preschool disabled children and ill health is highly likely, but heterogeneity had a detrimental effect on the precision of the pooled estimates, leading to wide predictive intervals which crossed zero in every analysis. Thus, caution must be observed in the generalization of the results of this review to other mother-caregiver populations.

Other known causes of heterogeneity were the inclusion of extreme SMDs and the limited study numbers, especially in the subgroup meta-analyses.

## Strengths and Limitations

The exclusion of abstracts and grey literature may have contributed to the apparent small study bias (Song et al. 2000). The screening, data extraction and quality assessment were performed by a single person and so some potentially eligible studies may have been missed (Edwards et al. 2002). The small number of studies limited the precision and generalizability of the findings, and the investigation of sources of heterogeneity (Higgins and Green 2011). The inclusion of only observational study designs risked exaggerated conclusions being drawn from biased studies or misattributed to caregiver burden when potentially influential factors were not measured, such as SES and child behavior (Bekhet et al. 2012; Biswas et al. 2015; Smith et al. 2001). The scoping approach to the range of symptoms and inclusion of stress identified variation in outcomes at unknown clinical levels. The inclusion of studies using different outcome measures introduced heterogeneity into both the overall and symptom subgroup meta-analyses. However, the outcome measures expected to identify greater stress in caregivers than other mothers due to measurement bias did not, in general, produce greater SMDs than those produced using other stress measures.

The use of predictive intervals illustrates the impact of heterogeneity of study designs and populations on estimates of association. The study identifies gaps in the understanding of factors that contribute to mother-caregiver health outcomes during the preschool period.

Caregiving is assumed to have a causal relationship to ill health due to the additional demands of the role (Raina et al. 2004). All the studies in this review were retrospective in design and only one (Laxman et al. 2015) enquired whether the symptom was present prior to or at the point of exposure (becoming a caregiver). Therefore, the direction of causality could not be inferred.

## Conclusions for Practice

Mothers with caregiving responsibilities for preschool children with developmental disabilities may have poorer health than those with typically developing children of the same age, although with high (unexplained) variability within and between caregiver populations. Investigation of the relationship of caregiving to general and physical ill health, and between stress and the development of clinically significant levels of adverse health during this period or later is warranted. If a causal relationship between caregiving and ill health is established (e.g. via assessment of health status prior to caregiving), investigation of factors known to influence caregiver health is needed to inform interventions to prevent and reduce ill health in mothers of preschool children with developmental disabilities.

## Electronic supplementary material

Below is the link to the electronic supplementary material.

Electronic supplementary material 1 (DOCX 40 kb)
